# Supplementary figures and images for: Hap10: reconstructing accurate and long polyploid haplotypes using linked reads
Source: BMC Bioinformatics. 2020 Jun 18;21:253. doi: 10.1186/s12859-020-03584-5 (PMC7302376; doi:10.1186/s12859-020-03584-5)

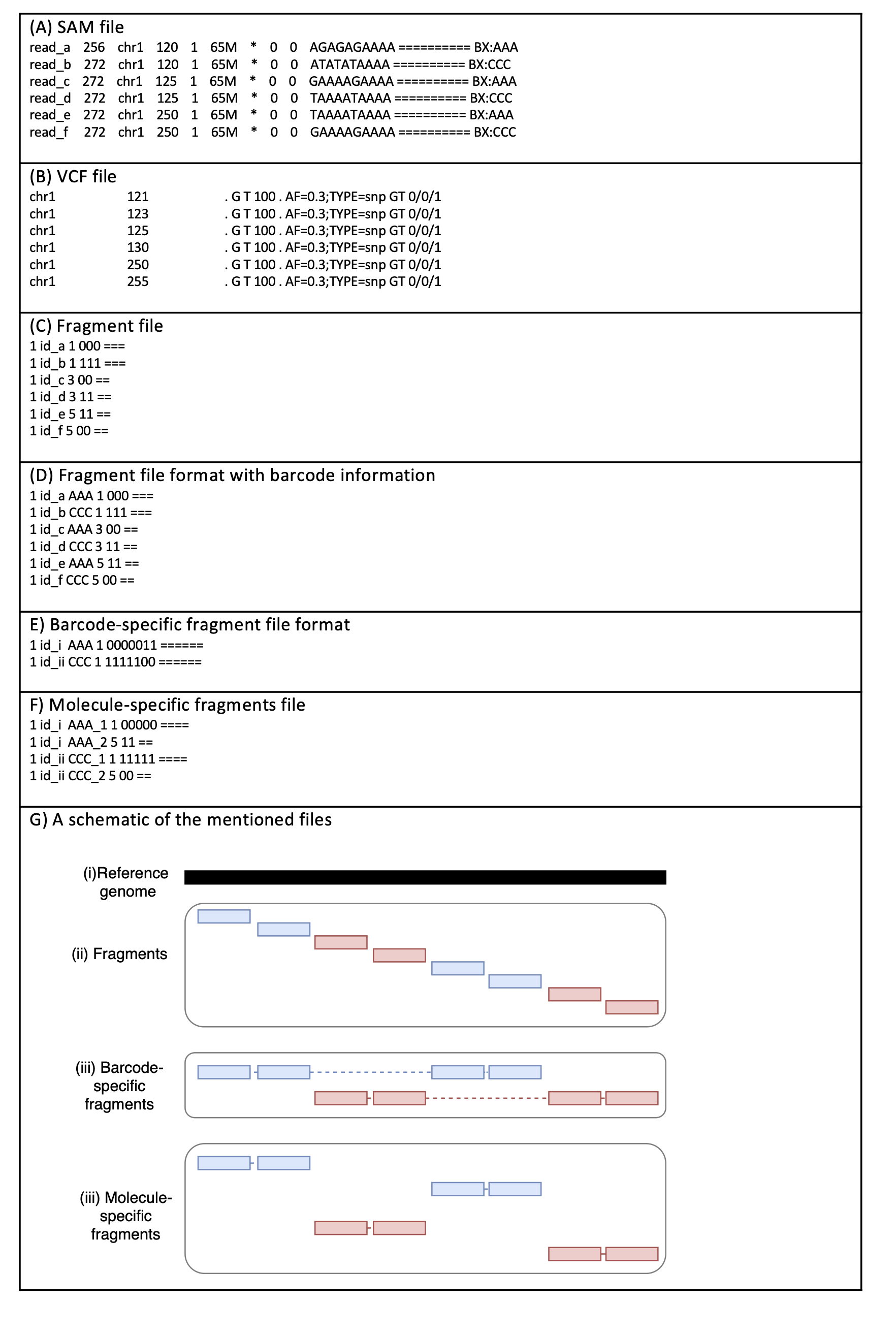

Supplement: Supplementary file 1 — Additional file 1: Figure S1. Description of the fragment file format using an example SAM and VCF input. A) In the SAM format, each line corresponds to a read (except for the header lines). The fourth column shows the genomic position of the first base of the aligned read. As a simple example, for each read 10 bases are shown in the 10th column. The next column shows the Phred quality of each base. Finally, the BX tag shows the barcode of each read provided by LongRanger software. For more information on the SAM format, see http://samtools.github.io. B) In the VCF format, the second column shows the genomic position of the variant in each line (except for the header lines). The third and fourth columns contain the reference and alternative alleles, respectively. The last column shows the genotype of the variant, in this example for a triploid. For more information on the SAM format see http://samtools.github.io. C) In fragment file designed for short reads, the first column shows the number of consecutive alleles (called part here) in the fragment, the second column the id of the fragment, the third column the start position of the first part, followed by the alleles of the part. The position is reported as the index of the variant in the VCF file, starting from 1. If there are more parts, they will appear next. The last column shows the Phred quality scores of all alleles in all parts consecutively. D) To include the barcode information for haplotyping, the barcodes in the SAM file BX tag are provided in the third column of the fragment file. The other columns are shifted accordingly. E) In Barcode-specific fragment file, reads with the same barcode are combined, as discussed in step one of "Hap++" Section. F) Molecule-specific fragments file is the output of step one of "Hap++" Section. The third column, which was the barcode, is iterated from one to the number of molecules for each barcode with an underscore in between. G) A schematic of the mentioned procedures [file 12859_2020_3584_MOESM1_ESM.jpg]

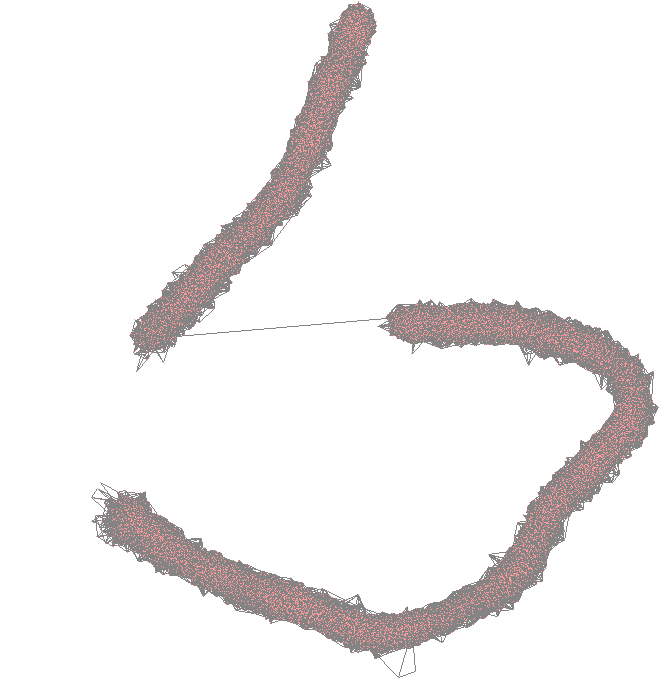

Supplement: Supplementary file 2 — Additional file 2: Figure S2. A graph indicating overlap between fragments. Red dots are vertices (corresponding to the fragments), grey lines are edges drawn when two fragments have at least one SNP in common. The depicted graph is for a case with 5 mb reference genome containing an N-region of 50 kb. The coverage is 15 per haploid and the SNP rate is 0.01. The average length of 10X DNA molecules for this simulation is set to 50 kb. Few fragments originate from a DNA molecule larger than 50 kb. The resulting graph has two separate subgraphs connected by a single edge. Note that one barcode-specific fragment connecting two read blocks is not sufficient for connecting the corresponding haplotypes. This phenomenon decreases the quality of reconstructed haplotype. The figure is generated using Cytoscape (www.cytoscape.org). [file 12859_2020_3584_MOESM2_ESM.jpg]
