# Supplementary material for: Hap10: reconstructing accurate and long polyploid haplotypes using linked reads
Source: BMC Bioinformatics. 2020 Jun 18;21:253. doi: 10.1186/s12859-020-03584-5 (PMC7302376; doi:10.1186/s12859-020-03584-5)
Supplement: Supplementary file 3 — Additional file 3: Table S1. An example of the haplotype output format. We report the reconstructed haplotypes as a text file with a specific format similar to that of HapCUT2. Each haplotype block starts with a line describing the length of the haplotype, number of reads corresponding to the block and the minimum error correction (MEC) score. From the next line, each row corresponds to each variant. The first and second columns show the 1-based index and variant position, respectively. Then, the next 2 ∗ ploidy columns are haplotypes and quality scores. For each allele of haplotypes, a quality score is provided. As a metric for quality, we use the number of matching reads at each position that are estimated for each haplotype. [file 12859_2020_3584_MOESM3_ESM.docx]

| Table S1. An example of the haplotype output format. We report the reconstructed haplotypes as a text file with a specific format similar to that of HapCUT2. Each haplotype block starts with a line describing the length of the haplotype, number of reads corresponding to the block and the minimum error correction (MEC) score. From the next line, each row corresponds to each variant. The first and second columns show the 1-based index and variant position, respectively. Then, the next $2*\mathrm{ploidy}$ columns are haplotypes and quality scores. For each allele of haplotypes, a quality score is provided. As a metric for quality, we use the number of matching reads at each position that are estimated for each haplotype. |
| --- |
| Block 1 Length of haplotype block 9 Number of read 58 Total MEC 10  1 644 0 0 1 2 9 8  2 783 0 0 1 10 13 15  3 916 0 0 1 9 16 13  4 990 0 0 1 11 12 16  5 1848 0 1 0 13 13 12  6 1862 1 0 0 13 13 14  7 1930 1 0 1 11 11 12  8 1042 0 0 1 13 14 16  9 1075 0 0 1 10 16 14 |
