# Supplementary material for: Hap10: reconstructing accurate and long polyploid haplotypes using linked reads
Source: BMC Bioinformatics. 2020 Jun 18;21:253. doi: 10.1186/s12859-020-03584-5 (PMC7302376; doi:10.1186/s12859-020-03584-5)
Supplement: Supplementary file 4 — Additional file 4: Table S2. The impact of the convergence threshold on Hap10 performance. A triploid genome of 230 kb with a SNP rate of 0.001 is simulated. The average molecule length and number of molecules per bead are 50 k and 10, respectively. [file 12859_2020_3584_MOESM4_ESM.docx]

| Table S2. The impact of the convergence threshold on Hap10 performance. A triploid genome of 230kb with a SNP rate of 0.001 is simulated. The average molecule length and number of molecules per bead are 50k and 10, respectively. | | | |
| --- | --- | --- | --- |
| **Conv. threshold** | **Reconstruction rate** | **Vector error rate** | **Time (sec.)** |
| $1$ | 0.790 | 0.10 | 27 |
| ${10}^{-2}$ | 0.826 | 0.19 | 45 |
| ${10}^{-8}$ | 0.941 | 0.06 | 50 |
