# Supplementary material for: Hap10: reconstructing accurate and long polyploid haplotypes using linked reads
Source: BMC Bioinformatics. 2020 Jun 18;21:253. doi: 10.1186/s12859-020-03584-5 (PMC7302376; doi:10.1186/s12859-020-03584-5)
Supplement: Supplementary file 5 — Additional file 5: Table S3. SDhaP with and without linked-read information. For the latter, the input data is considered as regular Illumina reads and barcodes are not used. The dataset is simulated using 1 Mb of chromosome one of potato genome with a SNP rate of 0.01. The coverage is 10. The results are averaged over 5 independent simulations. For the third row, we split the 1 Mb region into three independent parts of the same size. The last two rows present results of Hap++ and Hap10 on the same data. [file 12859_2020_3584_MOESM5_ESM.docx]

| Table S3. SDhaP with and without linked-read information. For the latter, the input data is considered as regular Illumina reads and barcodes are not used. The dataset is simulated using 1 Mb of chromosome one of potato genome with a SNP rate of 0.01. The coverage is 10. The results are averaged over 5 independent simulations. For the third row, we split the 1 Mb region into three independent parts of the same size. The last two rows present results of Hap++ and Hap10 on the same data. | | | |
| --- | --- | --- | --- |
| **Method** | **Avg. haplotype block length (no. SNPs)** | **Reconstruction rate** | **Vector error rate** |
| SDhaP  (without linked-read inf.) | 11.8 | 0.87 | 0.0004 |
| SDhaP  (with linked-read inf.) | 6213.4 | 0.71 | 0.200 |
| SDhaP (subregions) | 1815.4 | 0.78 | 0.023 |
| Hap++ | 2073.4 | 0.79 | 0.009 |
| Hap10 | 2074.6 | 0.84 | 0.007 |
