# Supplementary material for: Hap10: reconstructing accurate and long polyploid haplotypes using linked reads
Source: BMC Bioinformatics. 2020 Jun 18;21:253. doi: 10.1186/s12859-020-03584-5 (PMC7302376; doi:10.1186/s12859-020-03584-5)
Supplement: Supplementary file 6 — Additional file 6: Table S4. Performance of SDhaP at different coverage levels, for a triploid genome with SNP rate of 0.001. The average molecule length and number of molecules per bead are 50 k and 10, respectively. The results are averaged over 5 independent simulations. [file 12859_2020_3584_MOESM6_ESM.docx]

| Table S4. Performance of SDhaP at different coverage levels, for a triploid genome with SNP rate of 0.001. The average molecule length and number of molecules per bead are 50k and 10, respectively. The results are averaged over 5 independent simulations. | | | |
| --- | --- | --- | --- |
| **Coverage** | **Avg. haplotype block length (no. SNPs)** | **Reconstruction rate** | **Vector error rate** |
| 25 | 627 | 0.630 | 0.330 |
| 50 | 627 | 0.702 | 0.108 |
| 100 | 627 | 0.695 | 0.030 |
